# Species-Specific Inactivation of Triosephosphate Isomerase from *Trypanosoma brucei*: Kinetic and Molecular Dynamics Studies

**DOI:** 10.3390/molecules22122055

**Published:** 2017-11-24

**Authors:** Alejandra Vázquez-Raygoza, Lucia Cano-González, Israel Velázquez-Martínez, Pedro Josué Trejo-Soto, Rafael Castillo, Alicia Hernández-Campos, Francisco Hernández-Luis, Jesús Oria-Hernández, Adriana Castillo-Villanueva, Claudia Avitia-Domínguez, Erick Sierra-Campos, Mónica Valdez-Solana, Alfredo Téllez-Valencia

**Affiliations:** 1 Faculty of Medicine and Nutrition, Juarez University of Durango State, Av. Universidad y Fanny Anitua S/N, Durango 34000, Mexico; avazquezraygoza@gmail.com (A.V.-R.); avitiaclaudia@gmail.com (C.A.-D.); 2 School of Chemistry, Pharmacy Department, National Autonomous University of Mexico, Mexico City 04510, Mexico; qfblucia@gmail.com (L.C.-G.); Ivelazquez00@hotmail.com (I.V.-M.); Piter_jo@comunidad.unam.mx (P.J.T.-S.); rafaelc@unam.mx (R.C.); hercam@unam.mx (A.H.-C.); franher@unam.mx (F.H.-L.); 3 Biochemistry and Genetics Laboratory, National Institute of Pediatrics, Ministry of Health, Mexico City 04534, Mexico; jesus.oria.inp@gmail.com (J.O.-H.); acastilloinp@gmail.com (A.C.-V.); 4 Faculty of Chemical Sciences, Juarez University of Durango State, Av. Artículo 123 S/N Fracc. Filadelfia, Gomez Palacio, Durango 35010, Mexico; ericksier@gmail.com (E.S.-C.); valdezandyval@gmail.com (M.V.-S)

**Keywords:** human African trypanosomiasis, triosephosphate isomerase, enzymatic kinetics, molecular dynamics simulation

## Abstract

Human African Trypanosomiasis (HAT), a disease that provokes 2184 new cases a year in Sub-Saharan Africa, is caused by *Trypanosoma brucei*. Current treatments are limited, highly toxic, and parasite strains resistant to them are emerging. Therefore, there is an urgency to find new drugs against HAT. In this context, *T. brucei* depends on glycolysis as the unique source for ATP supply; therefore, the enzyme triosephosphate isomerase (TIM) is an attractive target for drug design. In the present work, three new benzimidazole derivatives were found as TbTIM inactivators (compounds **1**, **2** and **3**) with an I_50_ value of 84, 82 and 73 µM, respectively. Kinetic analyses indicated that the three molecules were selective when tested against human TIM (HsTIM) activity. Additionally, to study their binding mode in TbTIM, we performed a 100 ns molecular dynamics simulation of TbTIM-inactivator complexes. Simulations showed that the binding of compounds disturbs the structure of the protein, affecting the conformations of important domains such as loop 6 and loop 8. In addition, the physicochemical and drug-like parameters showed by the three compounds suggest a good oral absorption. In conclusion, these molecules will serve as a guide to design more potent inactivators that could be used to obtain new drugs against HAT.

## 1. Introduction

Human African Trypanosomiasis (HAT), also known as sleeping sickness, is part of the tropical neglected diseases group. It is a painful and prolonged suffering ailment, in which people often die if they do not get the appropriate treatment. The World Health Organization reported 2184 new cases, with approximately 70 million people at risk in 2016 [1]. Actually, only four drugs are used in the sleeping sickness treatment, melarsoprol, pentamidine, suramin, and, recently, the combined therapy of eflornitine/nifurtimox (NECT) [2]. The treatment is ineffective because it has limitations ranging from poor efficacy, acute toxicity, and parasite resistance [3,4,5,6,7]. Therefore, there is an urgency to develop new drugs against HAT.

HAT is a parasitic disease caused by the protozoan *Trypanosoma brucei*, which is transmitted by the bite of flies from the *Glossina* genus [8]. The parasite proliferates extracellularly in the mammalian bloodstream, and it has been demonstrated that the glycolysis is essential for survival as the only source for ATP supply [9]. Thus, the glycolytic enzymes are attractive targets mainly for their principal role in the energy production in parasites. In this context, the glycolytic enzyme triosephosphate isomerase (TIM) has been proposed by different research groups as a validated target for drug design against *T. brucei* [10,11,12].

Triosephosphate isomerase (E.C. 5.3.1.1) catalyzes the interconversion between glyceraldehyde-3-phosphate and dihydroxyacetone phosphate in the fifth step of the glycolytic pathway [13]. Structurally, TIM from *T. brucei* (TbTIM) is a homodimeric enzyme, and each monomer consists of 250 residues forming eight parallel β-strands surrounded by eight α-helices, showing the classical TIM barrel folding [10]. The active site is formed by Lys13, His95, and Glu167, the key residues for catalysis [14]; however, Asn11 has been reported as an important residue [15]. The interface between monomers occupies a significant portion of the molecular surface area of each monomer, approximately 1530 Å^2^ for TbTIM [16]. Similar to other isoforms, TbTIM is active only in its dimeric form, and several reports have shown that the use of small molecules to target the dimer interface induce structural modifications leading to enzyme inactivation [11,17,18,19]. With respect to TbTIM, there are only two reports about enzyme inactivation [11,17], the other two referring to enzyme inhibition [20,21]. 

On the other hand, in the search for new drugs, one of the important privileged scaffolds is the benzimidazole nucleus [22] because it has shown different biological activities such as cell antiproliferative effects [23], antioxidant properties [24], antiviral [25], antimicrobial [26], antitumor [27], anti-HIV [28], antiparasitic [29], anti-obesity and anti-diabetic [30].

In this context, benzimidazole derivatives synthetized by our group have shown biological activity against several parasites [31,32,33,34,35], and some of them have shown inactivation of *Trypanosoma cruzi* TIM (TcTIM) [36,37,38]. In the present work, we searched TbTIM inactivators from our *in house* library of benzimidazole derivatives. Three compounds were able to inactivate selectively the TbTIM, and their inactivation mechanism was characterized through enzyme kinetics and molecular dynamics simulations.

## 2. Results and Discussion

### 2.1. TbTIM Inactivation

**Figure 1:**
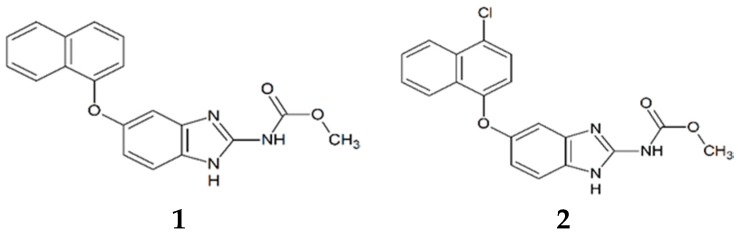

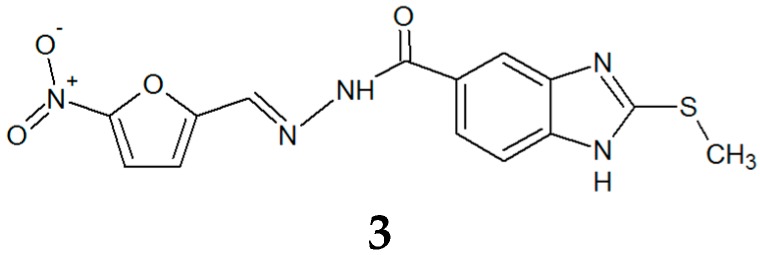
Structure of TbTIM inactivators.

In order to find hits for the development of new drugs against HAT, our *in house* library of 200 benzimidazole derivatives was tested against TbTIM, as described in the Materials and Methods section. From 200 molecules assessed, only three were able to inactivate TbTIM more than 50%, twenty-four less than 50% and up to 20%, and the rest under 20%. According to these data, the three most potent were selected for kinetic and structural characterization studies (compounds **1**, **2**, and **3**, Figure 1).

**Figure 2:**
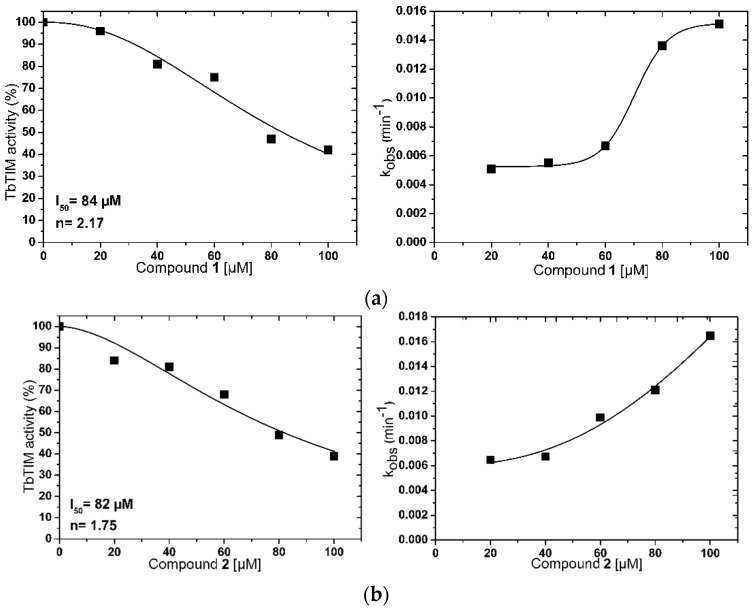

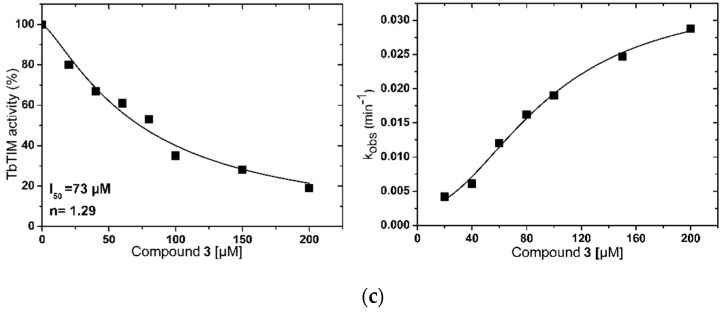
Activity of TbTIM (left panel) and plots of the pseudo-first-order rate constants (right panel) at different concentrations of compounds (**a**) **1**; (**b**) **2** and (**c**) **3**. I_50_ value was defined as the concentration of compound needed to reduce the enzymatic activity to 50% and determined through curves at different compound concentrations and a Hill coefficient, *n*, is a measure of the degree of cooperativity of the ligands.

Kinetic studies showed that compounds inactivated the enzyme in a concentration-dependent manner obtaining I_50_ values of 84, 82, and 73 µM for compounds **1**, **2** and **3**, respectively (Figure 2). Something interesting to highlight is that the inactivation curves of compounds **1** and **2** showed a sigmoidal decay behavior (Figure 2a,b), although the curves did not reach 100% of inactivation. This is supported by the *n* value close to two obtained in both cases. This suggests that the enzyme inactivation is a cooperative process, which involves at least two molecules of each compound [17], whilst compound **3** showed a hyperbolic behavior with an *n* value close to 1, suggesting that only one molecule was necessary for enzyme inactivation (Figure 2c). This behavior was similar to TcTIM inactivators reported previously [11,17,19]. Additionally, inactivation of TbTIM was reported in the same concentration range to that found here.

To continue with characterization, the pseudo-first-order rate constant (k_obs_) was obtained and plotted against the respective compound concentration to calculate the apparent second-order-rate constant (k_2app_) (Figure 2d–f). Moreover, due to solubility problems, a complete curve could not be generated; therefore, it was not possible to determine the value of k_2app_. Nevertheless, an interesting observation from the curves is that, in all cases, there was a tendency to saturation. This suggests that the inactivation velocity was dominated by the equilibrium between the association and dissociation of enzyme-compound complex [39], which means that, at high compound concentrations, the rate of inactivation will not change. This pattern was observed for TcTIM inactivators even using different types of molecules such as benzothiazole derivatives [17] or natural products [19].

**Figure 3:**
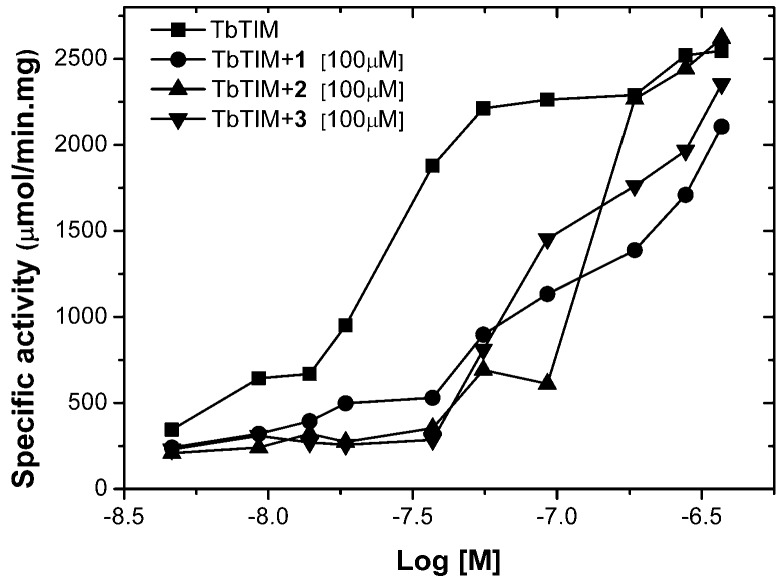
Effect of compounds **1**, **2** and **3** at different concentrations of TbTIM.

In order to continue kinetic characterization and take into account that TbTIM is active only in its dimeric form, it was considered important to determine if compounds act interfering in the association and dissociation process between monomers, which means if compounds bind into the dimer interface. To this end, assays at different enzyme concentrations and a fixed compound concentration were carried out. The results showed that, in the three cases, when the enzyme concentration increases, the effect of compound diminishes, suggesting that compounds **1**, **2**, and **3** are binding at the dimer interface of TbTIM (Figure 3). In the same way, this behavior was observed in TcTIM inactivators [17,19,21,40].

### 2.2. Molecular Dynamics

**Figure 4:**
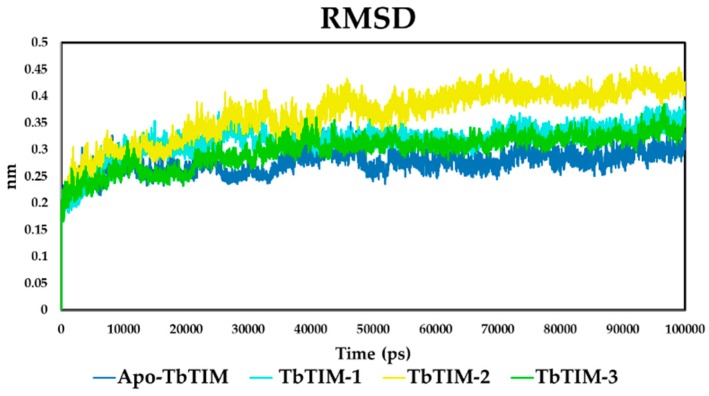
Root mean square deviation (RMSD) of free enzyme (Apo-TbTIM) and the TbTIM-compound complexes.

After kinetics studies, molecular dynamics simulations of 100 ns were performed to characterize the interaction between TbTIM and compounds **1**, **2**, and **3**. In the case of **1** and **2** and in accordance with that observed in kinetic data, two molecules of each compound were docked into the TbTIM interface, whilst, for compound **3**, only one was used. Firstly, the system stability was evaluated by means of RMSD (root mean square deviation) value. For each MD simulation, the first 10 ns were discarded as the equilibration period from further analysis. Then, the RMSD showed that the four systems, free enzyme (Apo-TbTIM), TbTIM-**1**, TbTIM-**2**, and TbTIM-**3**, obtained an RMSD of 0.3 nm among Cα from initial to final structure conformation, supporting that the system was stable during the simulation time (Figure 4). Moreover, a fact worth highlighting is the significantly different behaviors observed in the RMSD trajectory in each TbTIM-compound complex, being the most remote to the Apo the TbTIM-**2** complex. This indicates that the three systems underwent conformational changes to some degree, a behavior observed in other TIM-ligand complexes such as TcTIM-1,2,6-thiadiazine derivatives [41] and TcTIM-benzothiazole derivative [42].

**Figure 5:**
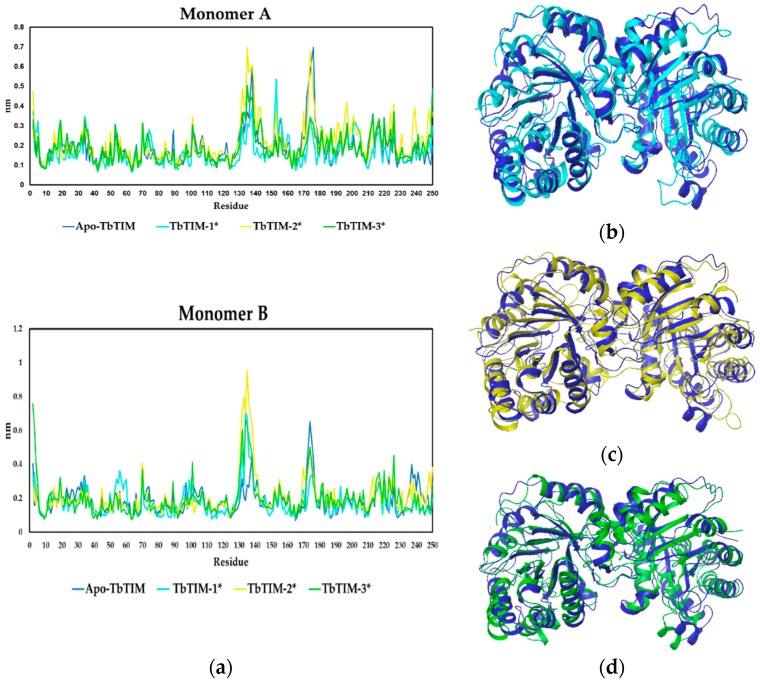
(**a**) Residue Mean-Square Fluctuations from monomer A and B (* *p* < 0.05). Superimposed average structures of Apo-TbTIM (blue) with TbTIM-**1** (**b**); TbTIM-**2** (**c**); and TbTIM-**3** (**d**). The alignments were made based on backbone.

Thereafter, to determine flexible regions in TbTIM structure, the Residue Mean-Square Fluctuations (RMSF) between Apo-TbTIM and each TbTIM-compound complex was carried out (Figure 5). The RMSF graphic showed differences between the Apo-TbTIM and complexes, a two-way ANOVA analysis with the null hypothesis that the ligand binding has no effect on the RMSF, yielded *p*-values of 0.013, 0.011, and 0.001 (<0.05) for compounds **1**, **2**, and **3**, respectively. This data rejects the null hypothesis and supports that the differences observed were because of ligand binding (Figure 5a). Moreover, based on backbone structure superposition, some clear differences were observed between the three complexes and Apo-TbTIM (Figure 5b–d).

#### 2.2.1. Structural Analyses of the TbTIM-Ligand Complex

In order to describe in detail the different conformations obtained along the 100 ns simulation, a clustering analysis based on size was performed with respect to RMSD. This takes into account the cluster with more structures in the same conformation and the main cluster of each group was selected for the binding ligand analysis.

**Figure 6:**
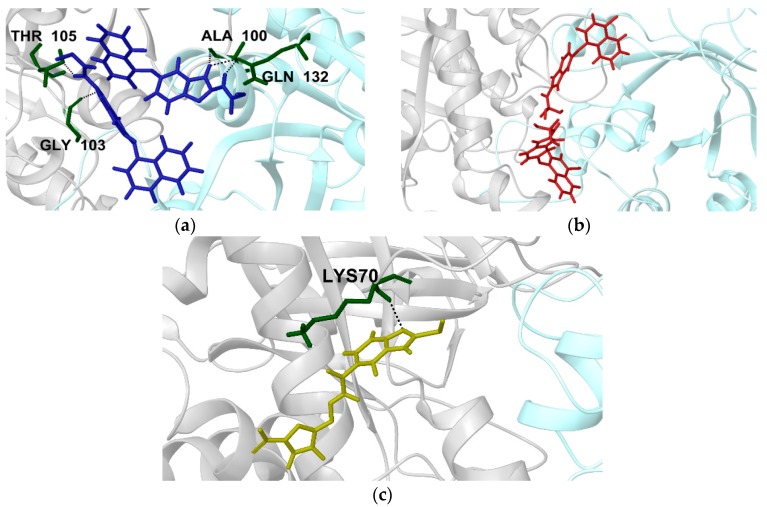
Binding mode of compounds **1**, **2** and **3** on TbTIM (Light Gray ribbons monomer A and Light turquoise ribbons monomer B. (**a**) two molecules of compound **1** (blue sticks); (**b**) two molecules of compound **2** (red sticks); and (**c**) compound **3** (yellow sticks). H-bonds are depicted as dotted lines.

As it was stated before, two molecules of compounds **1** and **2** were docked on the TbTIM interface (Figure S1a,b). For compound **1**, one molecule formed hydrogen bonding with Gly103 and Thr105 from monomer A, whilst the other molecule showed hydrogen bonding with Gln132 and Ala100 from monomer B (Figure 6a). In the case of compound **2**, for both molecules docked, no hydrogen bonds (H-bond) were established with any of monomers (Figure 6b). In fact, during 65% of MD simulation, no hydrogen bonds were detected; however, the two molecules were stable during the simulation. In relation to compound **3** (Figure S1c), the molecule showed a hydrogen bond with Lys70 from monomer A (Figure 6c) with a prevalence of 40% through MD. These data agreed with the average of H-bond found in MD analysis (Table 1). Similar binding modes have been observed for TcTIM inactivators [19,42,43,44,45]. The results emphasize the importance of the hydrophobic tunnel, which is formed at the dimer interface, where different types of ligands can be bound for the enzyme inactivation [11].

Additionally, Linear Interaction Energy (LIE) analysis revealed that the binding energy of compounds **1** and **2** was dominated by the van der Waals component, whilst, in compound **3**, it was dominated by the electrostatic interaction energy (Table 1). It is worth highlighting that, in order to obtain the (V_CL_)_free_ and (V_LJ_)_free_ values in compounds **1** and **2**, the average of the energy between the two molecules bounded was calculated.

#### 2.2.2. TbTIM Loop 6 and Loop 8 Dynamics

The TIM is a perfectly evolved enzyme formed by two monomers, each one with an independent catalytic site [14]. Moreover, neither cooperativity nor allosterism has been observed between the two active sites [18,46]. Nevertheless, the importance of certain domains such as loop 6 or catalytic loop (Glu168-Pro178) and loop 8 (Gly235-Lys240) has been reported [47,48,49,50,51]. For this reason, it was decided to carry out an analysis by clustering specifically in these regions along the MD simulations.

**Figure 7:**
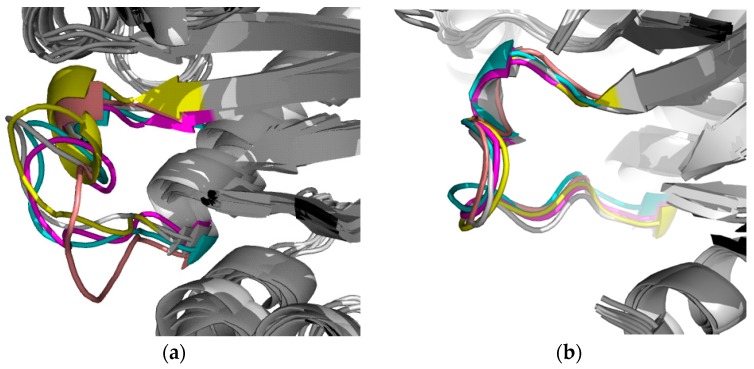

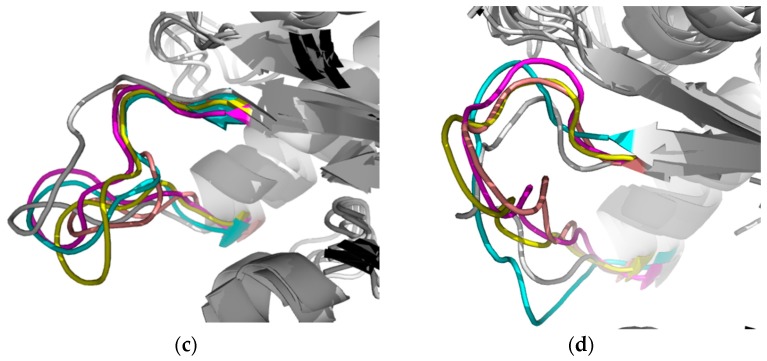
Catalytic loop 6 dynamics in (**a**) Apo-TbTIM; (**b**) TbTIM-**1**; (**c**) TbTIM-**2**; and (**d**) TbTIM-**3**. Cluster 1 (gray), Cluster 2 (cyan), Cluster 3 (magenta), Cluster 4 (yellow) and Cluster 5 (pink). The alignments were made based on backbone.

The importance of conformational dynamics and flexibility of loop 6 to carry out the catalysis has been reported, and it closes on the substrate and protects it from exposure to the solvent [52]. Specifically, the closed state of loop 6 during the enzyme-substrate complex formation is stabilized by the interaction between the phosphate-loop gripper phase and the phosphodianion of the substrate [53]. Here, a possible conformational change was observed between the Apo-TbTIM and the complexes, as was suggested by the differences in RMSF of the residues that are part of the loop (Figure S2a). The apparent formation of an α-helix was detected in Apo-TbTIM enzyme and in TbTIM-**1** complex, being more evident in the Apo-TbTIM form. In contrast, no possible secondary structure formation was presented in TbTIM-**2** and TbTIM-**3** complexes (Figure 7). Therefore, our data suggest that the perturbation in the dynamics of this domain observed with the inactivators could explain in part the enzyme inactivation.

**Figure 8:**
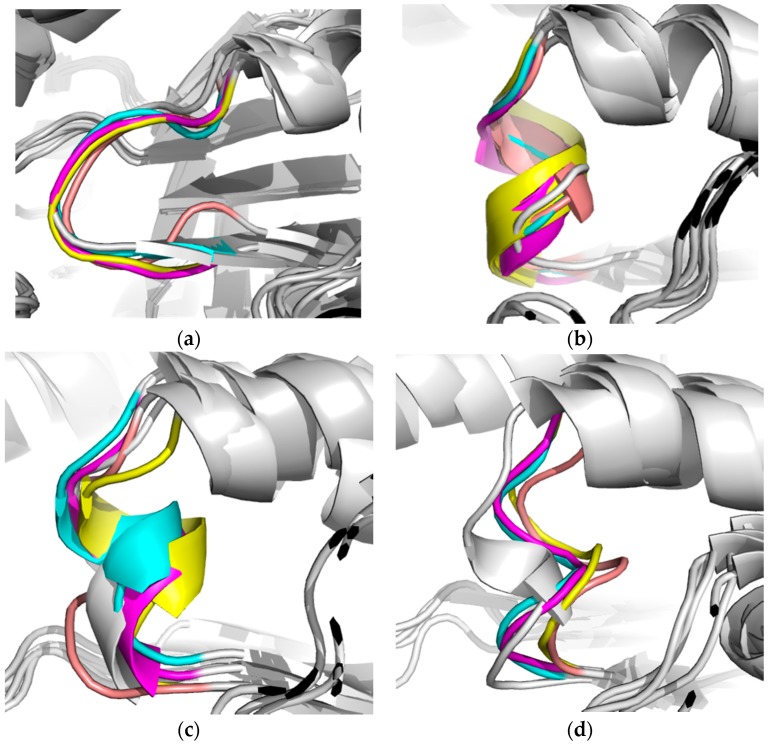
Loop 8 dynamics in (**a**) Apo-TbTIM; (**b**) TbTIM-**1**; (**c**) TbTIM-**2**; and (**d**) TbTIM-**3**. Cluster 1 (gray), Cluster 2 (cyan), Cluster 3 (magenta), Cluster 4 (yellow) and Cluster 5 (pink). The alignments were made based on backbone.

On the other hand, loop 8 analysis showed that, in the three complexes, as well as in loop 6, there were possible conformational changes (Figure S2b), more evident with compounds **1** and **2**, suggesting the formation of an α-helix (Figure 8a–c). TbTIM-**3** complex had more movement. This is because of side chain fluctuations, which led to the site instability (Figure 8d).

**Figure 9:**
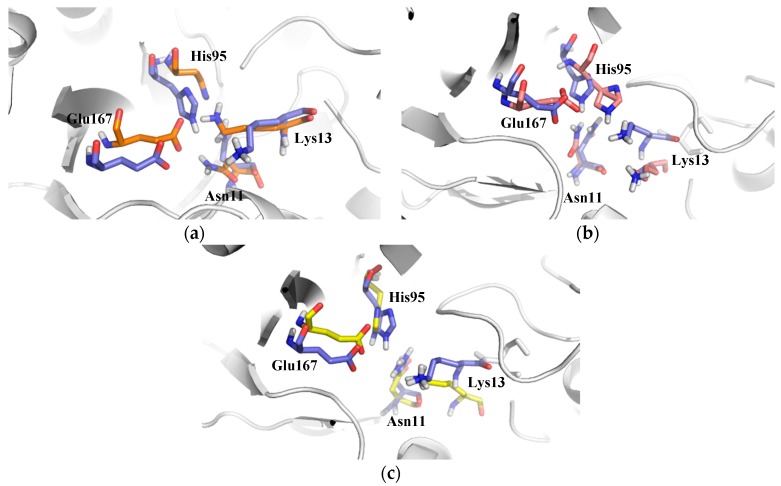
Movement of side chains from catalytic site residues in the Apo-TbTIM (blue sticks) and in complex with (**a**) compound **1**; (**b**) compound **2**; and (**c**) compound **3**.

It has been described that loop 6, loop 7, and loop 8 contribute to the active site geometry through H-bond interactions with the substrate [52]. Therefore, we decided to analyze and compare this region in the main cluster of the Apo-TbTIM with the three complexes. The results showed that there were differences in the geometry of the side chain in residues at the catalytic site (Figure 9a–c). Despite the compounds being bound at the dimer interface, and not interacting directly with catalytic residues, these changes indicated that the binding of compounds modify the global conformation of the protein, such that there was a change in the flexibility and conformational dynamics of the enzyme, suggesting that probably this conformational change entails the loss of the enzyme activity. Taking together all the data described above, it can be stated that the binding of compounds **1**, **2** and **3** into the TbTIM interface affected the conformational dynamics of the enzyme, and these changes could be responsible for the loss of enzymatic activity observed in the in vitro assays.

### 2.3. Effects of Compounds on Human Triosphosphate Isomerase (HsTIM)

**Figure 10:**
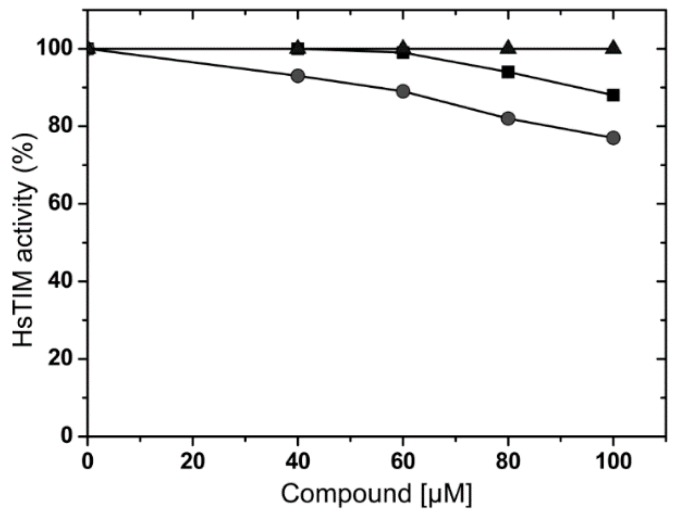
Inactivation of HsTIM by compounds **1** (squares), **2** (circles) and **3** (triangles).

When a therapeutic target is present in both the pathogen and in the host, a desirable characteristic is the selectivity of the inhibitors or inactivators. To this end, we studied the effects of these molecules in the HsTIM. Compounds were tested at the highest possible concentration, which depended on individual solubility. Results showed that molecules **1** and **2** inactivated 12% and 23% HsTIM, respectively, when they were tested at 100 µM (Figure 10). At this concentration, TbTIM lost 77% of its activity (Figure 2). Compound **3** was tested until 200 µM (data not showed) and no effect on HsTIM activity was observed, whilst TbTIM lost 80% at the same concentration (Figure 2). Therefore, these compounds are selective for TbTIM with respect to the human counterpart.

### 2.4. In Silico Analysis of ADME-Tox Properties

An important issue to address was the Administration, Distribution, Metabolism, and Excretion (ADME) properties and the possible toxicological effects of these compounds. Therefore, several in silico studies were made through different software available on the web (Table 2). With respect to ADME properties, data suggested that the three compounds bearing physicochemical characteristics to be considered as potential drug candidates. Toxicological analysis revealed that the predicted LD50, estimated in rodents, was closer to 1 g/Kg in the three cases, suggesting no potential toxicological effects.

## 3. Materials and Methods

### 3.1. Synthesis and Molecular Characterization

Compounds **1** and **2** were prepared from the appropriate o-phenylendiamine and carrying out our synthetic procedure previously reported for similar benzimidazole derivatives [31]. Compound **3** was synthesized beginning with 2-(methylthio)-1*H*-benzimidazole-5-carboxylic acid by a three reaction sequence: (1) preparation of tert-butyl 2-(2-(methylthio)-1*H*-benzimidazole-5-carbonyl)hydrazinecarboxylate, as intermediate, using tert-butyl carbazate, COMU ((1-cyano-2-ethoxy-2-oxoethylidenaminooxy)dimethylamino-morpholino-carbenium hexafluorophosphate), NMM (4-methylmorpholine) in CH_2_Cl_2_ at rt; (2) hydrolysis of protecting group with TFA (trifluoroacetic acid); and (3) reaction with 5-nitrofuran-2-carbaldehyde in CHCl_3_ and acetic acid in catalytic amounts at rt for 12 h.

*Methyl(5-(naphthalen-1-yloxy)-1H-benzimidazol-2-yl)carbamate* (**1**). Purified by MeOH washes. m.p. 227.6–229.8 °C. 1H NMR (DMSO-*d*_6_; 400 MHz) δ: 3.73 (s, 3H, –OCH_3_), 6.82 (d, *J* = 7.6 Hz, 1H, H-2′), 6.90 (dd, *J* = 8.6, 2.4 Hz, 1H, H-6), 7.08 (d, *J* = 2.3 Hz, 1H, H-4), 7.41 (t, *J* = 7.9 Jz, 1H, H-3′), 7.43 (d, *J* = 8.6 Hz, 1H, H-7), 7.52–7.61 (m, 2H, H-6′,H-7′), 7.65 (d, *J* = 8.3 Hz, 1H, H-5′), 7.96 (d, *J* = 7.6 Hz, 1H, H-4′), 8.20 (d, *J* = 9.0 Hz, 1H, H-8′), 11.64 (s, 2H, –NH). ^13^C NMR (DMSO-*d_6_*; 100 MHz) δ: 52.5, 104.6, 111.8, 113.2, 114.6, 121.6, 122.5, 125.8, 126.1, 126.2, 126.8, 127.9, 134.6, 148.0, 151.5, 154.0, 154.8. MS (EI *m*/*z*) (%ab): 333 (M^+^, 72%); 301 (M − 32, 100%); 272 ([301 − 29], 21%); 127 ([272 − 145], 16%).

*Methyl(5-((4-chloronaphthalen-1-yl)oxy)-1H-benzimidazol-2-yl)carbamate* (**2**). Purified by MeOH washes. m.p. 248.7–253.5 °C. 1H NMR (DMSO-*d_6_*; 400 MHz) δ: 3.74 (s, 3H, –OCH_3_), 6.76 (d, *J* = 8.3 Hz, 1H, H-2′), 6.92 (dd, *J* = 8.5, 2.4 Hz, 1H, H-6), 7.14 (d, *J* = 2.3 Hz, 1H, H-4), 7.45 (d, *J* = 8.5 Hz, 1H, H-7), 7.58 (d, *J* = 8.3 Hz, 1H, H-3′), 7.69 (ddd, *J* = 8.1, 6.9, 1.1 Hz, 1H, H-7′), 7.77 (ddd, *J* = 8.4, 6.9, 1.2 Hz, 1H, H-6′), 8.19 (d, *J* = 8.4 Hz, 1H, H-5′), 8.33 (d, *J* = 8.3 Hz, 1H, H-8′), 11.68 (s, 2H). ^13^C NMR (DMSO-*d*_6_; 100 MHz) δ: 52.5, 105.0, 111.3, 113.4, 114.6, 122.3, 123.8, 123.9, 126.3, 126.6, 127.0, 128.4, 130.8, 148.0, 150.7, 153.7, 154.7. MS (EI *m*/*z*) (%ab): 369 (M + 2, 33%), 367 (M^+^, 46%); 335 (M − 32, 100%); 299 ([335 − 36], 22%); 272 ([299 − 27], 21%).

*(E)-2-(Methylthio)-N′-((5-nitrofuran-2-yl)methylene)-1H-benzimidazole-5-carbohydrazide* (**3**). Purified by chromatographic column using CHCl_3_/MeOH (95:5) as eluent. m.p. 202.1 °C with decomposition. 1H NMR (DMSO-*d*_6_; 400 MHz) δ: 2.74 (s, 3H, S–CH_3_), 6.69 (bs, 1H, N–H), 7.24 (d, *J* = 4 Hz, 1H, H-3′), 7.56 (d, *J* = 12 H, 1H, H-7), 7.76 (m, 2H, H-6, H-4′), 8.06 (s, 1H, H-4), 8.41 (s, 1H, N=C–H), 12.23 (s, 1H, CON-H). ^13^C NMR (DMSO-*d*_6_; 100 MHz) δ: 14.3 (double signal), 113.7 (double signal), 115.1 (double signal), 122.5, 126.7, 135.4, 138.9, 141.7, 152.3, 152.4, 154.9, 158.6, 159.1, 164.1.

### 3.2. Expression and Purification of TIMs

The proteins TbTIM and HsTIM were expressed in *Escherichia coli* and purified as described in the literature [54], obtaining a 95% of purity measured by densitometric analysis. After purification, enzymes were dissolved in 100 mM triethanolamine, 10 mM EDTA, and 1 mM DTT (pH 8). Then, they were precipitated with (NH_4_)_2_SO_4_ (80% saturation) for storage at 4 °C. Before use, exhaustive dialysis against 100 mM triethanolamine, 10 mM EDTA (pH 7.4) was performed. Protein concentration was determined by absorbance at 280 nm using a molar extinction coefficientof 34,950 and 33,460 M^−1^·cm^−1^ for TbTIM and HsTIM, respectively.

### 3.3. Enzymatic Activity Assays

Enzymatic activity was determined in an enzyme-coupled reaction using α-glycerol phosphate dehydrogenase, in the direction of glyceraldehyde 3-phosphate (GAP) to dihydroxyacetone phosphate, as reported elsewhere [11,17,19]. The decrease in absorbance at 340 nm was followed in a multicell Agilent (Santa Clara, CA, USA) spectrophotometer at 25 °C. The reaction mixture contained 100 mM triethanolamine, 10 mM EDTA, 0.2 mM NADH, 1 mM GAP, and 90 ng/mL of α-glycerol phosphate dehydrogenase (α-GPDH) (pH 7.4). The reaction was initiated by addition of 5 ng/mL either TbTIM or HsTIM.

### 3.4. Inactivation Assays

The enzyme was incubated at 5 µg/mL in 100 mM triethanolamine, 10 mM EDTA (pH 7.4), 10% (*v*/*v*) DMSO, and the compound at the indicated concentration at 36 °C for 2 h. After this, aliquots of the mixture were withdrawn to perform the activity assay above mentioned. The initial screening was perfomed testing our in-house chemical library (200 compounds), at a concentration of 200 µM. The I_50_ value (concentration of the compound needed to reduce the enzymatic activity to 50%) was determined through curves at different compound concentrations and adjusting the data to the equation reported elsewhere [17].

Enzyme inactivation velocity was determined by a pseudo first order rate constant (k_obs_), measuring activity at different times and a fixed compound concentration, adjusting the data to Equation (1)

(1) $$A\;{=\;A_{0}e}^{-\mathrm{kt}},$$

where *A* is the activity at the indicated compound concentration, *A*_0_ is the activity in the absence of compound, *k* is the pseudo-first order constant, and *t* is the time.

### 3.5. Molecular Docking

Ligand parameters, such as torsions, atom types and atomic partial charges of Gasteiger-Marsilli [55], were generated using AutoDock Tools (ADT) 4.2 (La Jolla, CA, USA) [56]. The protein structure was obtained from the Protein Data Bank with PDB-ID 2J27 [57]. Protein was prepared adding hydrogen and atomic partial charges of Gasteiger using ADT 4.2. Docking simulations were performed using AutoDock 4.0 (La Jolla, CA, USA) [58] and the Lamarckian genetic algorithm with a grid size of *X* = 82, *Y* = 40 and *Z* = 42 points, centered at the dimer interface. One hundred runs of the genetic algorithm were performed for each ligand–receptor pair using the default parameters. The output ligand configurations were clustered and the best binding energy scores were selected for the molecular dynamics simulations.

### 3.6. Molecular Dynamics

Ligands parameterizations were performed in a PRODRG server [59] with a GROMOS87 force field (Biomos b.v., Laboratory of Physical Chemistry, Zürich, Switzerland) [60]. All simulations were carried out using the GROMOS9643a1 force field [61]. At the begining, an energy minimization using 500 cycles of the steepest-descent algorithm was executed. The first velocities were assigned according to the Maxwell distribution to 10 K temperature and it was gradually increased to 300 K. After that, we carried out a canonical (NVT) [62] and isothermal-isobaric (NPT) [63] simulations (with isotropic position scaling) at 300 K and 1 atm pressure, using a truncated cubic periodic box with dimensions of 100 Å, filled with Simple point charge water model [64] and neutralized with Na^+^ and Cl^−^ ions. Finally, we performed a complete molecular dynamics simulation of 100 ns at 300 K without restrictions using the software GROMACS 5.1 (Groningen Biomolecular Sciences and Biotechnology Institute, Groningen, The Netherlands) [65], obtaining 10,000 conformations that were saved every 5000 steps.

### 3.7. Binding Energy

The calculation of the binding free energy was based on the linear interaction energy (LIE) method by the Equation (2):

∆G_bind_ = α[(V_LJ_)_bound_ − (V_LJ_)_free_] + β[(V_CL_)_bound_ − (V_CL_)_free_] + γ (2)

where (V_LJ_)_bound_ is the average Lennard–Jones energy for ligand–protein interaction; (V_LJ_)_free_ is the average Lennard–Jones energy for ligand–water interaction; (V_CL_)_bound_ is the average electrostatic energy for ligand–protein interaction; (V_CL_)_free_ is the average electrostatic energy for ligand–water interaction; α, β, and γ are the LIE coefficients. For small drug-like ligands, α = 0.18, β = 0.50 and γ = 0.00 [66,67,68]. 

### 3.8. Toxicological and Physicochemical Properties

The Faf-Drugs4 server [69] was used for calculating relevant drug-like properties and the web server PROTOX [70] was used to predict LD50.

## 4. Conclusions

Three new benzimidazole derivatives were found with the capacity to inactivate selectively the TbTIM with respect to the HsTIM, and with the characteristics to be considered as potential drug candidates. Therefore, these molecules will serve as a guide for the design of more potent inactivators that could be used to obtain new drugs against HAT.

## Figures and Tables

**Table 1:** Binding free energies calculated by the Linear Interaction Energy method and hydrogen bonds of the complexes during molecular dynamics.

Complex	Energy (kcal/mol)					Hydrogen Bond	
	(V_LJ_)_bound_	(V_LJ_)_free_	(V_CL_)_bound_	(V_CL_)_free_	∆G_bind_	Range	Average
TbTIM-1	−29.45	−6.53	−4.45	−1.28	−5.71	0–7	2
TbTIM-2	−38.90	−2.67	−0.92	−0.33	−6.82	0–6	0
TbTIM-3	−27.52	−8.49	−44.83	−43.59	−4.05	1–7	1

(VLJ)_bound_ average Lennard–Jones energy for ligand–protein interaction; (VLJ)_free_ is the average Lennard–Jones energy for ligand–water interaction; (VCL)bound is the average electrostatic energy for ligand–protein interaction; (VCL)_free_ is the average electrostatic energy for ligand–water interaction.

**Table 2:** Physicochemical and toxicological properties of TbTIM inactivators.

Compound	cLogP ^1^	tPSA ^1^	HB-D ^1^	HB-A ^1^	VEBER Rules ^1^	EGAN Rules ^1^	Predicted LD50 ^2^ (mg/kg)
1	4.1256	76.24	2	6	Good	Good	911
2	5.3882	53.08	1	6	Good	Good	990
3	1.9686	154.4	2	9	Good	Good	1000

^1^ Obtained in FAFDrugs4 server; ^2^ Calculated in PROTOX-Prediction of Rodent Oral TOXicity server. cLogP, logarithm of the partition coefficient between n-octanol and water; tPSA, topological polar surface area; HB-D, hydrogen bond donors; HB-A, hydrogen bond acceptors.

## Supplementary Materials

The supplementary materials are available online. Figure S1: Complete view of the binding mode of compounds (**a**) **1**; (**b**) **2**; and (**c**) **3**, at the dimer interface of TbTIM predicted by docking. For compounds **1** and **2**, the two molecules are displayed. Molecular surface is drawn as a purple grid, Figure S2: RMSF comparison between Apo-TbTIM and the complexes with compounds **1**, **2** and **3**. (**a**) Loop 6 residues; (**b**) Loop 8 residues.
