# Supplementary material for: Species-Specific Inactivation of Triosephosphate Isomerase from Trypanosoma brucei: Kinetic and Molecular Dynamics Studies
Source: Molecules. 2017 Nov 24;22(12):2055. doi: 10.3390/molecules22122055 (PMC6149853; doi:10.3390/molecules22122055)
Supplement: Supplementary file 1 [file molecules-22-02055-s001.pdf]

Supplementary Material

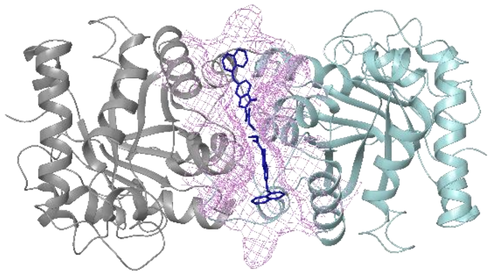

(**a**)

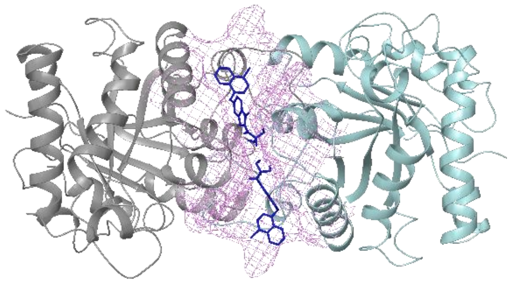

(**b**)

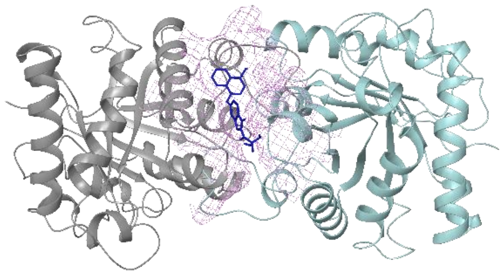

(**c**)

Figure S1. Complete view of the binding mode of compounds (**a**) **1**, (**b**) **2**, and (**c**) **3**, at the dimer interface of TbTIM predicted by docking. For compounds **1** and **2** the two molecules are displayed. Molecular surface is drawn as a purple grid.

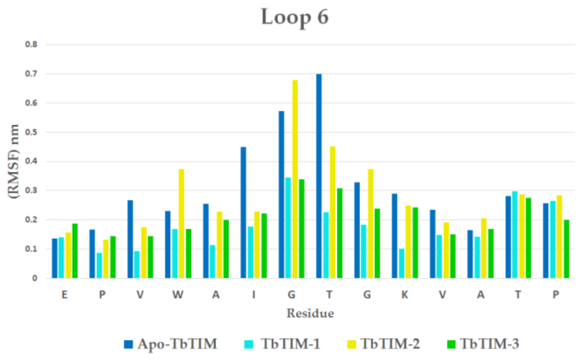

(a)

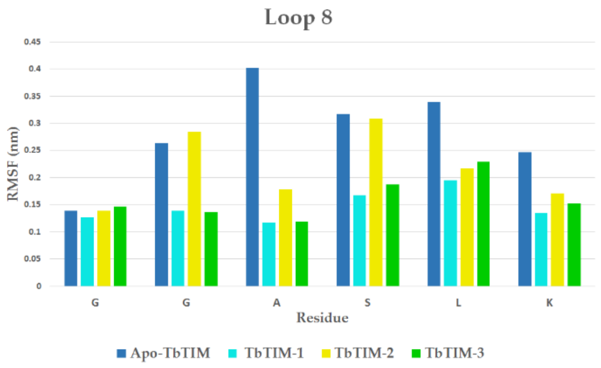

(b)

Figure S2. RMSF comparison between Apo-TbTIM and the complexes with compounds **1**, **2** and **3**. (**a**) Loop 6 residues. (**b**) Loop 8 residues.
